# Complete remission of *Helicobacter pylori*-negative gastric MALT lymphoma following eradication therapy: A case report

**DOI:** 10.1097/MD.0000000000050407

**Published:** 2026-08-21

**Authors:** Hui Chen, Jingtong Tang, Lin Mo, Xi Wang, Guobin He

**Affiliations:** a Department of Gastroenterology, Affiliated Hospital of North Sichuan Medical College, Nanchong, Sichuan, China; b Department of Pathology, Affiliated Hospital of North Sichuan Medical College, Nanchong, Sichuan, China.

**Keywords:** complete remission, eradication therapy, gastric MALT lymphoma, *Helicobacter pylori*-negative, non-*H pylori* helicobacters

## Abstract

**Rationale:**

Gastric mucosa-associated lymphoid tissue (MALT) lymphoma is typically associated with *Helicobacter pylori* infection; however, approximately 10% of cases are *H pylori*-negative. Management guidelines differ between the European Society for Medical Oncology (ESMO) and the National Comprehensive Cancer Network (NCCN) regarding first-line treatment for these patients. Here, we report a case of *H pylori*-negative gastric MALT lymphoma that achieved complete remission following standard eradication therapy.

**Patient concerns:**

A woman of Asian ethnicity in her late 50s presented with a 4-month history of intermittent, left-upper-quadrant abdominal pain. The patient had no significant medical or family history of gastric cancer or lymphoma.

**Diagnoses:**

Upper gastrointestinal endoscopy revealed irregular whitish mucosa with ill-defined borders in the distal gastric body. Biopsies demonstrated dense lymphoid infiltrates positive for CD20, CD79a, CD43, kappa light chain, focal CD23, and CD21, with a Ki-67 index of approximately 8%, consistent with extranodal marginal zone lymphoma (MALT lymphoma). *H pylori* testing by ^13^C-urea breath test (DOB 3.5; cutoff < 4.0) and Giemsa staining was negative, although the DOB value was close to the cutoff threshold. Positron emission tomography-computed tomography and endoscopic ultrasound confirmed Lugano stage IE disease confined to the mucosa and submucosa.

**Interventions:**

After multidisciplinary consultation, the patient received a 14-day bismuth-containing quadruple eradication regimen consisting of bismuth potassium citrate (2.4 g) twice daily, esomeprazole (40 mg) twice daily, amoxicillin (1 g) twice daily, and clarithromycin (0.5 g) twice daily.

**Outcomes:**

At 2-month follow-up, the patient was asymptomatic. Positron emission tomography-computed tomography showed no abnormalities, and repeat endoscopy with biopsies demonstrated complete histological remission, which persisted at 6-month follow-up.

**Lessons:**

This case adds to the growing evidence that *H pylori* status alone should not preclude a trial of eradication therapy in patients with localized gastric MALT lymphoma. Potential mechanisms include the role of non-*H pylori* helicobacters, the direct antitumor effects of antibiotics, and immunomodulation. Larger prospective studies are needed to identify predictors of response in this population.

## 1. Introduction

Gastric mucosa-associated lymphoid tissue (MALT) lymphoma is an indolent B-cell neoplasm that accounts for approximately 5% of all non-Hodgkin lymphomas, typically affecting adults aged 50 to 60 years.^[1]^ While the majority of gastric cases are associated with *Helicobacter pylori* infection, evidence indicates that approximately 10% of gastric MALT lymphomas are *H pylori*-independent.^[2]^ Recent studies have reported an increasing incidence of *H pylori*-negative gastric MALT lymphomas.^[3]^

The current management guidelines exhibit transatlantic divergence. In accordance with the European Society for Medical Oncology (ESMO) guidelines, *H pylori* eradication therapy is recommended as an initial treatment strategy regardless of the patient’s *H pylori* infection status.^[4]^ In contrast, the National Comprehensive Cancer Network (NCCN) continues to position radiotherapy as the standard first-line treatment for *H pylori*-negative localized disease.^[5]^ This discrepancy reflects the ongoing uncertainty regarding the biological mechanisms underlying *H pylori*-negative cases and reported complete remission (CR) rates following eradication therapy, ranging from 15% to 64% in meta-analyses.^[6]^ Here, we report a case of *H pylori*-negative gastric MALT lymphoma with comprehensive immunohistochemical documentation that achieved CR after standard quadruple eradication therapy. This case illustrates a clinical scenario consistent with an ESMO-guided approach and adds to the literature by suggesting the potential role of non-*H pylori* helicobacters (NHPHs) in Asian populations. However, as a single case report, it is hypothesis-generating rather than confirmatory.

## 2. Case report

**Figure 1.**
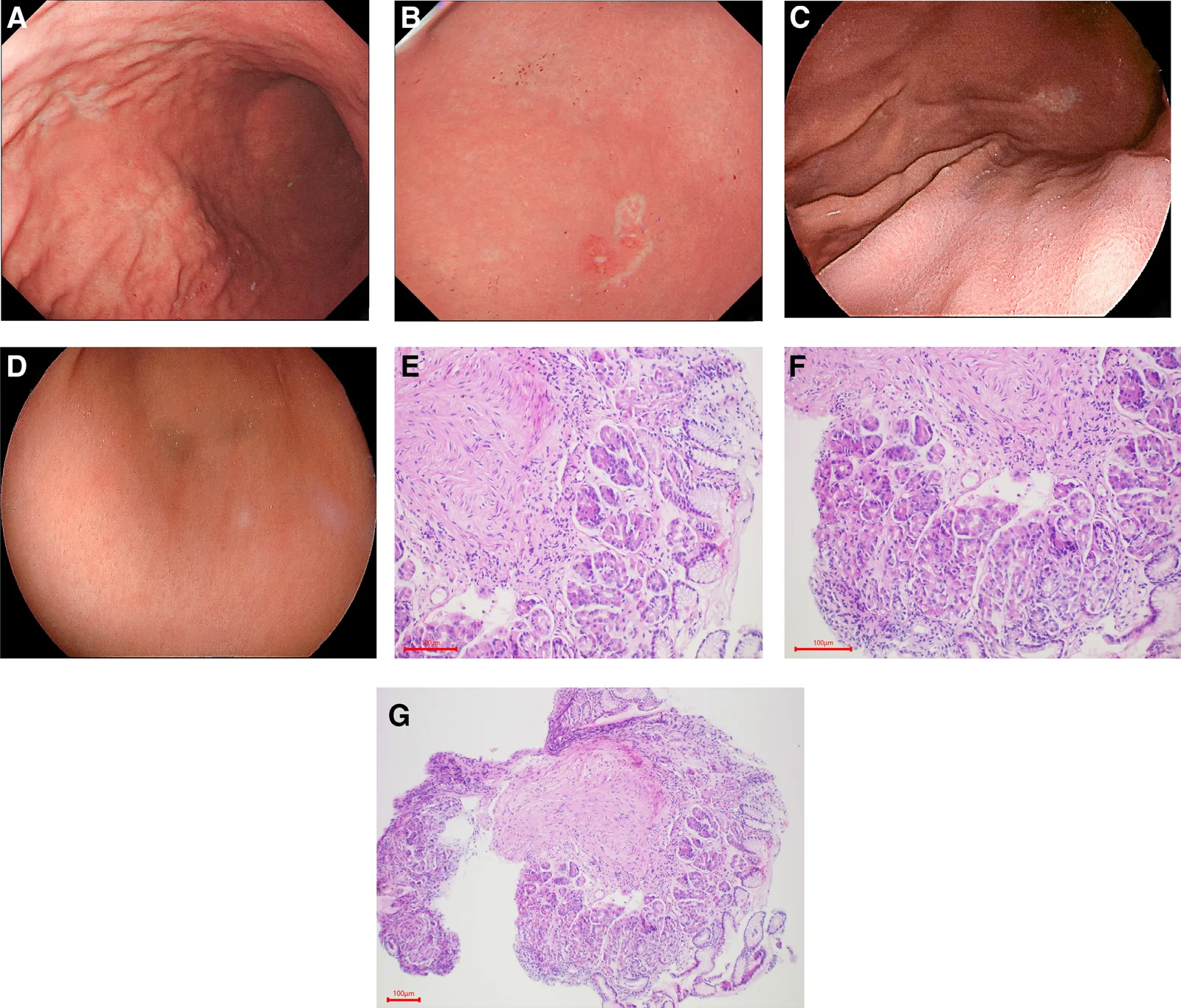
Endoscopic appearance before and after treatment and posttreatment histopathological findings. (A, B) Initial endoscopy (September 12, 2024) showing focal irregular mucosa with whitish discoloration and ill-defined borders in the distal gastric body. (C, D) Follow-up endoscopy at 2 months (November 2024) showing a residual patchy whitish mucosa at the body-antrum junction. (E–G) Posttreatment histopathological examination (hematoxylin and eosin staining, original magnification ×100, ×200, and ×400) showing mild chronic inflammation of the mucosa with no evidence of atrophy, intestinal metaplasia, activity, or definitive neoplasia (GELA score, complete remission). GELA = Groupe d’Etude des Lymphomes de l’Adulte.

**Figure 2.**
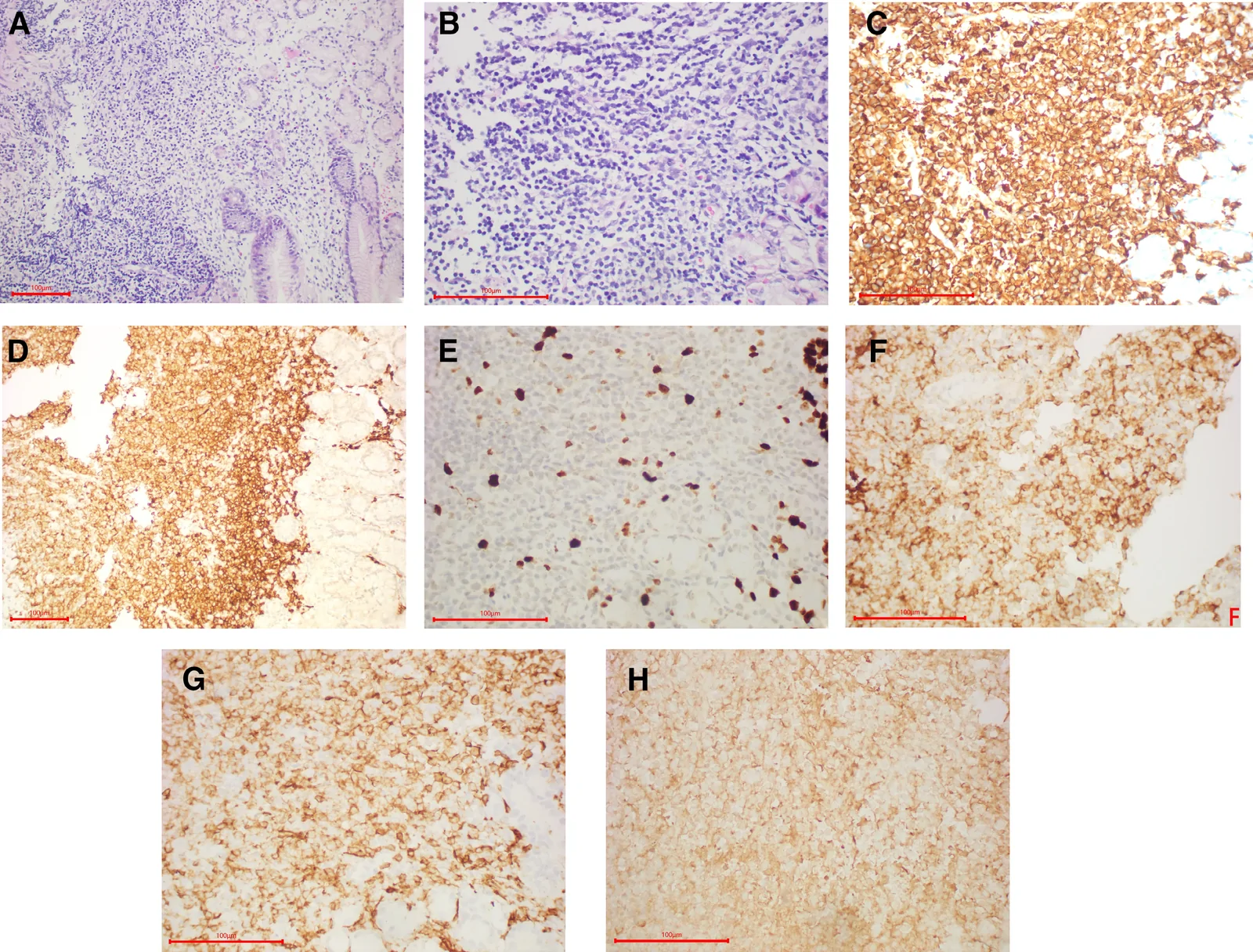
Histopathological and immunohistochemical features at diagnosis. (A, B) Hematoxylin and eosin (H&E) staining (original magnification ×100 and ×200) reveals dense lymphocytic infiltration within the mucosal lamina propria. (C–H) Immunohistochemical staining (original magnification ×200) shows positive expression for CD20 (C), CD79a (D), Ki-67 (approximately 8%; E), focal CD23 (F), CD43 (G), and kappa light chain (H). Staining for CD3, CD5, cyclin D1, lambda light chain, and CD10 was negative (data not shown).

A woman of Asian ethnicity in her late 50s presented to our endoscopy unit on September 12, 2024 with a 4-month history of intermittent, left-upper-quadrant abdominal pain. She had no significant medical or family history of gastric cancer, lymphoma, or nonsteroidal anti-inflammatory drug use. Endoscopic findings: Upper gastrointestinal endoscopy revealed a focal area of irregular mucosa with whitish discoloration and ill-defined borders in the distal gastric body (Fig. 1A, B). The surrounding mucosa appeared normal without atrophy or intestinal metaplasia. Histopathological and immunohistochemical biopsy specimens showed dense lymphoid infiltrates within the lamina propria (Fig. 2A, B). Immunohistochemical staining revealed that the infiltrate was positive for CD20 (Fig. 2C), CD79a (Fig. 2D), CD43 (Fig. 2G), and kappa light chain (Fig. 2H) with focal expression of CD23 (Fig. 2F) and CD21. The Ki-67 proliferation index was approximately 8% (Fig. 2E). Cells were negative for CD3, CD5, cyclin D1, lambda light-chain, and CD10. These findings were indicative of gastric extranodal marginal zone lymphoma (MALT lymphoma) with an indolent phenotype. The differential diagnosis included other low-grade B-cell lymphomas such as follicular lymphoma and mantle cell lymphoma; however, the absence of CD10 and cyclin D1 expression, respectively, helped exclude these entities. *H pylori* status assessment: A ^13^C-urea breath test result was negative (DOB value, 3.5; cutoff < 4.0). The patient confirmed that she had not taken proton pump inhibitors or antibiotics 4 weeks prior to testing, and no *H pylori*-like organisms were identified on histopathological examination using Giemsa staining. Although the DOB value was close to the cutoff threshold, raising the possibility of a false-negative result, the patient was classified as *H pylori*-negative based on the convergence of both negative test results. Molecular tests for *H pylori* and NHPHs, such as PCR, were not performed because of limited tissue availability. Staging workup: Contrast-enhanced computed tomography of the chest, abdomen, and pelvis, as well as positron emission tomography/CT (PET-CT), revealed no evidence of nodal, splenic, or distant extranodal involvement. Endoscopic ultrasound demonstrated that the lesion was confined to the mucosa and submucosa without infiltration beyond the muscularis propria. On the basis of these findings, the disease was classified as Lugano stage IE. A bone marrow biopsy was not performed, given the localized nature of the disease on imaging and the absence of peripheral blood abnormalities. Treatment: Following multidisciplinary consultation with hematology, and after thorough discussion of the potential benefits and uncertainties of eradication therapy for *H pylori*-negative disease, the patient elected to receive a 14-day bismuth-containing quadruple therapy regimen: bismuth potassium citrate 2.4 g twice daily, esomeprazole 40 mg twice daily, amoxicillin 1 g twice daily, and clarithromycin 0.5 g twice daily. The patient completed the entire treatment course without any adverse events. The clinical timeline of this case is summarized in Table 1.

**Table 1:** Clinical timeline of the case.

Date	Event	Key findings
September 12, 2024	Initial diagnosis	Endoscopy and biopsy confirmed stage IE *H pylori*-negative gastric MALT lymphoma
September 2024	Treatment	14-d bismuth-containing quadruple eradication therapy
November 2024	2-mo follow-up	Complete clinical, endoscopic, and histological remission
March 2025	6-mo follow-up	Sustained remission, no evidence of recurrence

MALT = mucosa-associated lymphoid tissue.

At the 2-month follow-up, the patient was completely asymptomatic. Surveillance abdominal computed tomography showed no abnormalities. PET-CT revealed no focal gastric wall thickening or abnormal fluorodeoxyglucose (FDG) avidity. Repeat endoscopy demonstrated a residual patchy whitish mucosa on the greater curvature at the body-antrum junction (Fig. 1C, D). Targeted biopsies from this area (Fig. 1E–G) showed only mild chronic inflammation, with no evidence of lymphomatous involvement, atrophy, intestinal metaplasia, or active *H pylori* infection. The patient was enrolled in a structured endoscopic surveillance program. To date (6-month follow-up), there has been no clinical, endoscopic, or histological evidence of disease recurrence or progression. Given the indolent nature of MALT lymphoma and the possibility of late relapse, continued long-term surveillance is required to confirm the durability of this remission.

## 3. Discussion

The pathogenesis of *H pylori*-negative gastric MALT lymphoma involves complex mechanisms including microbial factors, genetic alterations, and autoimmune components.^[7]^ In this case, the patient’s dramatic response to eradication therapy, confirmed by PET-CT, endoscopy, and histology, warrants a discussion of potential underlying mechanisms.

First, regarding microbial factors, in patients with *H pylori*-negative MALT lymphoma, the potential role of other gastric bacteria cannot be overlooked. These include *Helicobacter suis*, *Helicobacter heilmannii*, *Helicobacter felis*, and *Helicobacter salomonis*, collectively referred to as NHPHs. NHPHs may release bacterial toxins that alter the function and number of parietal and G cells, thereby influencing gastric acid secretion.^[8]^ During the infection process, fluctuations in gastric acid secretion, whether decreased or increased, may promote colonization by gastric Clostridium species. This colonization can contribute to gastric epithelial cell death, and the resulting microbial metabolites may facilitate ulcerogenesis. Furthermore, prolonged exposure of the gastric mucosa to inflammatory stimuli can lead to erosion, ulceration, and potentially neoplastic transformation through the inflammation-metaplasia-dysplasia cascade.^[9]^ Although we hypothesize that the eradication of coexisting NHPHs may have contributed to the remission observed in our patient, we acknowledge that this remains speculative in the absence of microbiological or molecular confirmation. An alternative, yet related, hypothesis involves the genetic features of the tumor itself. Second, from a genetic perspective, chromosomal translocations are well-recognized alterations in gastric MALT lymphoma lymphomagenesis. The most common are *t*(11;18)(q21;q21), *t*(1;14)(p22;q32), *t*(14;18)(q32;q21), and *t*(3;14)(p14.1;q32). These translocations lead to constitutive activation of the NF-κB pathway, promoting cell survival and proliferation.^[10]^ In our patient, molecular testing for *t*(11;18) was not performed due to limited tissue availability, and thus the translocation status remains unknown. We acknowledge this as a limitation of our case report. Notably, *t*(11;18) positivity is generally associated with a lower response rate to antibiotic therapy; however, without direct testing, we cannot infer the translocation status from the patient’s clinical response. Third, the direct antitumor and immunomodulatory effects of antibiotics, particularly clarithromycin, may also play a role. Clarithromycin has been shown to possess immunomodulatory properties that may suppress lymphoproliferation.^[11]^ Additionally, in the context of autoimmune diseases, chronic inflammatory stimulation of B cells by autoimmune complexes downregulates the regulatory effect of tumor necrosis factor alpha-induced protein 3 (TNFAIP3) on the NF-κB pathway, leading to excessive B-cell proliferation and ultimately contributing to malignant transformation.^[12]^ A meta-analysis by Jung et al demonstrated that patients with *H pylori*-negative gastric MALT lymphoma confirmed by 2 or more diagnostic tests can still achieve CR following eradication therapy, with a pooled CR rate of 32%.^[6]^ Our patient’s case adds to the growing body of evidence that *H pylori* status alone should not preclude a trial of eradication therapy in patients with localized gastric MALT lymphoma, even when the negative status is based on tests with a potential for false-negative results.

Our findings should be interpreted in the context of several important limitations. First, although the patient tested negative by both ^13^C-UBT and histology, the possibility of a false-negative *H pylori* result cannot be entirely excluded, particularly given the borderline DOB value (3.5) close to the cutoff (4.0). Second, we lacked microbiological or molecular confirmation (e.g., species-specific PCR) of NHPH infection in this patient, and thus the role of NHPH in mediating the response to therapy remains hypothetical. Third, the 6-month follow-up period was relatively short for indolent lymphomas such as MALT, and longer surveillance is required to confirm the durability of remission. Fourth, the absence of *t*(11;18) translocation testing prevents a more complete assessment of the tumor’s biological behavior.

In conclusion, *H pylori* eradication therapy is noninvasive, well-tolerated, and may be reasonably considered a first-line therapeutic option, even in carefully selected patients with *H pylori*-negative gastric MALT lymphoma. If symptoms persist after eradication, or in cases with distant metastasis or persistent lymphocytic infiltration on follow-up histology, additional treatments, including immunotherapy, radiotherapy, or surgery, should be pursued. The long-term durability of remission in such patients must be confirmed through continued endoscopic surveillance.

## Acknowledgments

The authors thank the Department of Pathology, Affiliated Hospital of North Sichuan Medical College, for their technical assistance with immunohistochemical staining.

## Author contributions

**Conceptualization:** Hui Chen.

**Data curation:** Hui Chen, Xi Wang.

**Formal analysis:** Hui Chen, Jingtong Tang, Xi Wang.

**Investigation:** Hui Chen.

**Methodology:** Hui Chen, Jingtong Tang, Guobin He.

**Resources:** Lin Mo.

**Supervision:** Guobin He.

**Validation:** Xi Wang.

**Writing – original draft:** Hui Chen.

**Writing – review & editing:** Guobin He.
